# Discovery and classification of complex multimorbidity patterns: unravelling chronicity networks and their social profiles

**DOI:** 10.1038/s41598-022-23617-8

**Published:** 2022-11-21

**Authors:** Javier Alvarez-Galvez, Esteban Vegas-Lozano

**Affiliations:** 1 Department of Biomedicine, Biotechnology and Public Health, University of Cadiz, Cadiz, Spain; 2 Department of Genetics, Microbiology and Statistics, University of Barcelona, Barcelona, Spain

**Keywords:** Diseases, Medical research, Risk factors

## Abstract

Multimorbidity can be defined as the presence of two or more chronic diseases in an individual. This condition is associated with reduced quality of life, increased disability, greater functional impairment, increased health care utilisation, greater fragmentation of care and complexity of treatment, and increased mortality. Thus, understanding its epidemiology and inherent complexity is essential to improve the quality of life of patients and to reduce the costs associated with multi-pathology. In this paper, using data from the European Health Survey, we explore the application of Mixed Graphical Models and its combination with social network analysis techniques for the discovery and classification of complex multimorbidity patterns. The results obtained show the usefulness and versatility of this approach for the study of multimorbidity based on the use of graphs, which offer the researcher a holistic view of the relational structure of data with variables of different types and high dimensionality.

## Introduction

The increasing number of individuals with co- and multimorbidities poses an urgent need to improve the management of patients with multiple coexisting diseases^1^. A better understanding of their causal mechanisms is needed to develop efficient early diagnosis, prevention and monitoring, as well as better tailored treatments for patients with and without multiple diseases across the lifespan. In addition, there are many different aetiological models of multimorbidity. The World Health Organisation (WHO) defines multimorbidity as "the presence of two or more chronic diseases in the same person"^2^. Likewise, the European Primary Care Research Network (EGPRN) proposes to translate the term multimorbidity into English as the "combination of a chronic disease with at least one other disease (acute or chronic), a biopsychosocial factor (associated or not) or a risk factor"^3^. In this sense, due to the complexity of pathogenesis and co-occurrence of chronic diseases, it is possible to find different variants of the concept of multimorbidity within the specialised literature^4^. In this work, seeking an operational approach at the population level and oriented towards prevention, we will define multimorbidity as the co-occurrence of two or more chronic diseases or long-term medical conditions in an individual^5–7^.

Multimorbidity is associated with lower quality of life, increased disability, functional impairment, increased health care utilisation and fragmentation of care, complex treatment and increased mortality^8,9^. Multimorbidity is expected to become even more prevalent in future years as chronic diseases gradually increase during the ageing process, resulting in increased disease persistence, functional disability, polypharmacy, health service use and mortality among the elderly. Indeed, the steady increase in the number of patients with complex clinical profiles due to the presence of chronic diseases as a consequence of the epidemiological transition represents a major challenge for everyday clinical practice, as well as for health systems and epidemiological research^10^. Today, patients with multiple chronic diseases are the rule rather than the exception^11^. In this sense, multimorbidity is one of the major global challenges facing governments and health systems^12^. In this field, one of the problems to be addressed is that health care is organised and oriented towards the treatment of individual diseases, but not so much towards the treatment of multiple pathologies that interact simultaneously^13^. Recent studies have highlighted the inability of current clinical guidelines to address the complex needs of patients with multimorbidity due to inadequate or modest attention to coexisting conditions^14,15^.

While multimorbidity is a global concern, most research efforts on physical and mental multimorbidity have focused on developed countries with advanced health systems^16^ where chronic pathologies are more prevalent due to the higher life expectancy. However, as indicated by Prados-Torres et al.^16^, the increase in multimorbidity is only partially explained by population ageing^4^. As recent work has shown^12^, multimorbidity is not exclusively limited to older people living in high-income countries (HICs). Multimorbidity also affects the young in our societies, and particularly those living in low- and middle-income countries (LMICs)^17^. The World Health Survey has estimated an average prevalence of multimorbidity of 7.8% in 28 LMIC^18^. Furthermore, more than one fifth of participants in six LMIC had multimorbidity^19^. Similarly, a recent study has shown among primary care patients almost all the elderly, half of the adults and one in 10 children have multimorbidity^20^. In this sense, other factors, mainly socio-economic, play an essential role in this trend, showing an advance of 10–15 years in the age of onset of multimorbidity among people with fewer socio-economic resources^21–23^. The degree and pattern of multimorbidity are particularly associated with social and demographic factors such as gender, age, educational level, income and, in general, any type of social inequality^1,12,16,24–28^. Recent work shows that multimorbidity is strongly associated with socioeconomic factors^21^ and this condition appears 10–15 years earlier in disadvantaged classes^1^.

### Current challenges in multimorbidity research

On the one hand, there is a need to provide new theoretical evidence to help improve prevention, diagnosis, prognosis, development of therapies and treatments and, ultimately, efficient monitoring of multimorbidity in clinical practice. Therefore, for a better understanding of multimorbidity it is essential: (1) to trace the complex network of factors linked to multimorbidity, both individual and contextual, and (2) to understand the complex causal mechanisms that explain the emergence and development of chronic diseases together. On the other hand, at the methodological-analytical level, there is a growing consensus on the need to increase and improve the quality of multimorbidity research through long-term prospective designs, new predictive methods, qualitative research for a better understanding of the problems associated with their quality of life and also better integration into clinical practice^29,30^, and also cost-effectiveness studies to ensure the sustainability of the health system in the face of the growing challenge of multimorbidity^4^. Therefore, new methodological strategies are needed to provide evidence that, in parallel, enable the development of new clinical practice guidelines (CPG). According with these gaps, as a first step new models are needed to identifying complex patterns of multimorbidity, i.e., new models that allow a better characterisation of multimorbidity as a health and social problem^31–34^.

In this context, the study of conditional independence relationships using undirected graphical models has recently emerged as a central technique for the statistical analysis of complex data with high dimensionality^35–42^. These models can be used for the estimation of the underlying graphical relationships in datasets that include variables from different domains, i.e. variables that compose different latent dimensions. Although there are methods for estimating Gaussian Graphical Models (GGMs), they usually have two drawbacks: firstly, there is a potential loss of information due to the necessary transformations and, secondly, they cannot incorporate categorical (nominal) variables. However, these problems have recently been overcome. In recent decades, MGMs have become a common way of representing complex systems and obtaining information about the relational patterns between observed variables in a wide variety of scientific disciplines such as statistical mechanics^35^, biology^36^, genetics^37^, neuroscience^38^ and psychology^39,40^. The mgm package^41^ uses the *glmnet* package^42^ to fit penalised generalised linear models (GLMs) to perform network neighbour selection^43–45^. The *mgm* package is geared towards estimating Mixed Graphical Models (MGMs) and Mixed Autoregressive Models (mVARs), both as stationary and time-varying models.

In this work, the aim is to explore complex relations between chronic conditions through a network-based approach. Although we know from previous studies (generally based on clustering techniques and latent class analysis) that there are well identified multimorbidity groups such as the cardiovascular or musculoskeletal ones, here we hypothesize that there are complex multimorbidity patterns that can be characterised through a disease community approach (i.e., focusing in disease neighbours that are commonly connected). Although no consensus definition of what is meant by complex multimorbidity has been found in the literature^46–48^, from here on we will understand it as patterns of high prevalence in several chronic conditions that are not clearly related and that, in general, have not been adequately characterized in the scientific literature. Specifically, we assess the application of Mixed Graphical Models (MGM) and community detection algorithms for the discovery and classification of complex networked multimorbidity patterns using large dataset from the Spanish European Health Survey (SEHS). On the one hand, we aim to (1) identify patterns (i.e., disease communities) of multimorbidity among the Spanish population by using MGM that will enable visual analysis of the pairwise relationships between predictors in network models, and on the other to (2) assess the combined use of MGM and community detection algorithms for the study and understanding of complex multimorbidity patterns.

## Results

### Descriptive analysis

**Figure 1:**
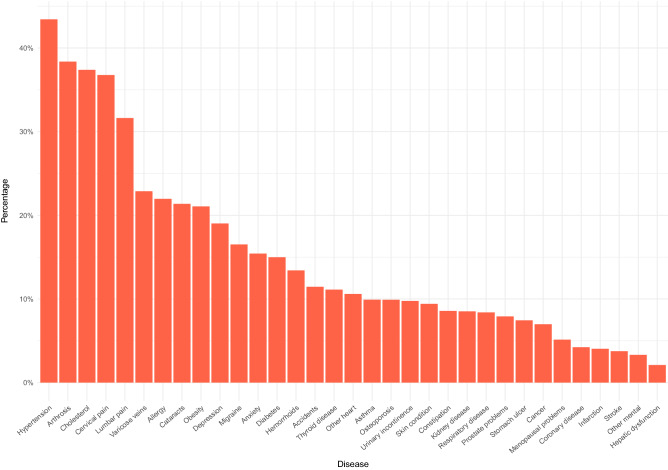
Percentages of diagnosed diseases.

The sample consisted of a total of 5002 men (40.7%) and 7,281 women (59.3%), with an average age of 60.8 years (SD = 17.0), having multimorbidity (i.e., two or more diagnosed chronic conditions), distributed across a total of 19 regions (or autonomous communities) in Spain. Figure 1 shows the response rates for each of the chronic disease diagnosis variables that would be used in the subsequent analyses for the composition of the multimorbidity patterns, among which the more prevalent were hypertension, arthrosis, lumbar back and cervical pain, and cholesterol.

### Mapping the complex structure of multimorbidity

The estimation algorithm of the MGM package requires us to make an assumption about the highest order interaction in the graph. Thus, in our case, it was assumed that there are, at most, pairwise interactions in the true graph, so the value of k = 2 was initially set. The algorithm includes an L1 penalty to obtain a sparse estimate that allows a better reading of the possible association structure. In addition, while there was a choice between selecting the lambda ($\lambda$) regularisation parameter using cross-validation (CV) or the extended Bayesian information criterion (EBIC), EBIC (with a gamma hyperparameter of $\gamma$ = 0.25) was finally selected which is slightly more conservative and it offered interpretable values while providing information on disease relationships that might be less clinically intuitive. Although the MGM library offers the option of using cross-validation to work on model fit, the exploratory nature of our work led us to opt for EBIC, as our study is eminently exploratory in nature for the identification of multimorbidity patterns. In other words, we did not seek to work on the optimal adjustment of the predictive model, but rather to identify those patterns that were relevant from a statistical point of view and, at the same time, interpretable from a clinical point of view.

**Figure 2:**
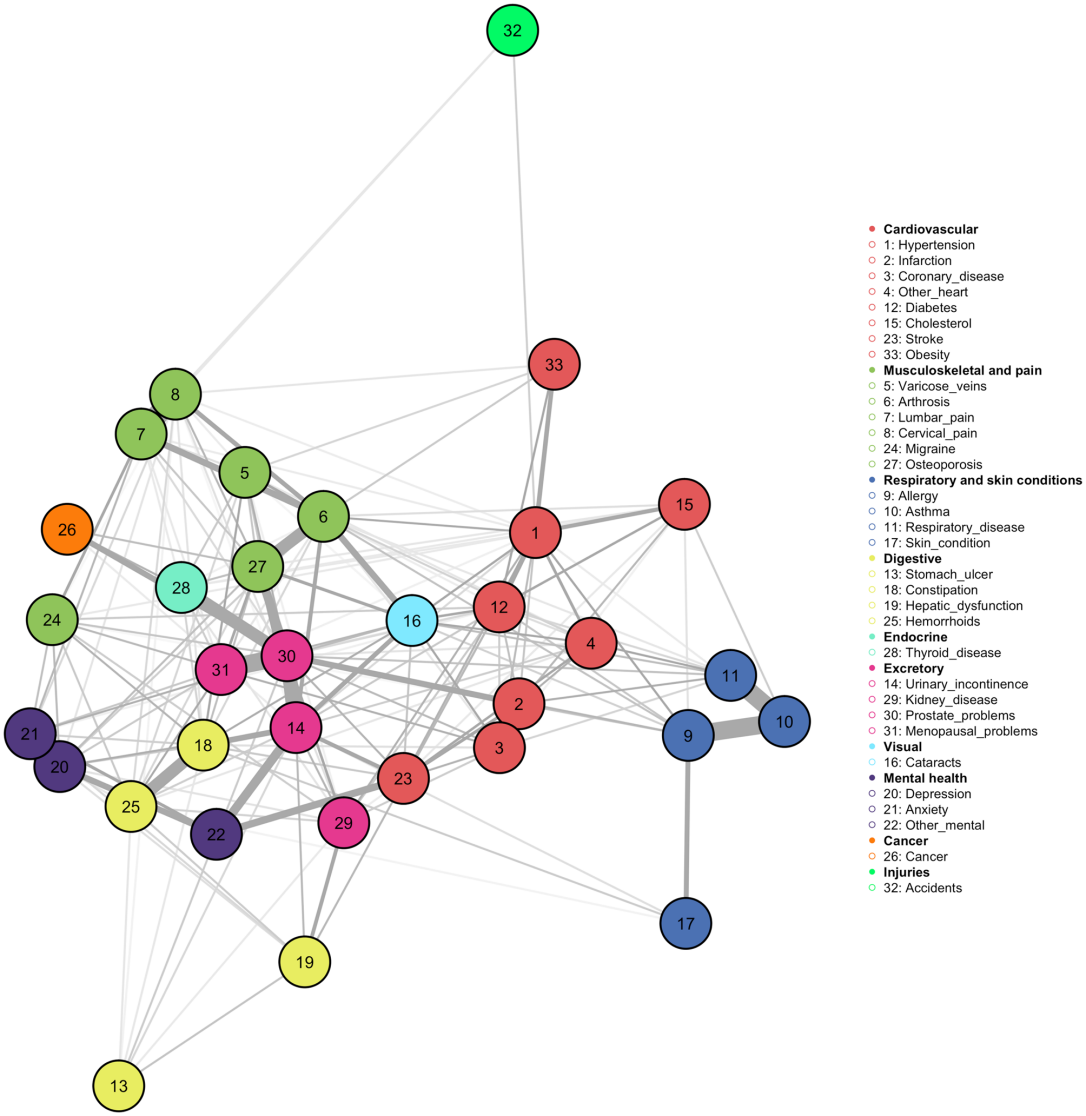
Mixed Graphical Model using the full dataset (k = 2).

Figure 2 below shows the first result of the MGM we implemented for the whole sample of people with multimorbidity (N = 12,283). As can be seen in the figure, the diagnosed chronic diseases were segmented into nine groups: (1) cardiovascular [hypertension, myocardial infarction, coronary heart disease, other heart disease, diabetes, cholesterol, stroke, obesity]; (2) musculoskeletal and pain [varicose veins, arthrosis, low back pain, neck pain, migraines, osteoporosis]; (3) respiratory and skin problems [allergy, asthma, respiratory problems, skin problems]; (4) digestive problems [stomach ulcer, constipation, liver dysfunction, haemorrhoids, and thyroid problem]; (5) excretory (or urinary) related [urinary incontinence, prostate problems, menopausal problems, and kidney disease]; (6) visual [cataracts]; (7) mental health [depression, anxiety, other mental health problems]; (8) cancer [malignant tumours]; and (9) accidents [disabling accidents].

As might be expected, the resulting graph identified some of the co-occurring disease relationships that had previously been identified in literature. Thus, at first glance, a high degree of closeness between diagnoses of cardiovascular diseases, mental health problems, allergy-related diseases and associated respiratory problems could be observed, while also providing information on other relationships that are less manifest in clinical practice, such as: (1) the relationship of cancer with back pain and migraines; (2) the connections between allergic and cardiovascular conditions; (3) the relationship of mental health problems with pain conditions and stomach ulcers, kidney and liver problems (which could be linked to the use of certain medications that might affect the function of certain organs); (4) the central role of excretory system problems and cataracts which, as shown, present a great transversality and connectivity between the different multimorbidity patterns; (5) the block of variables that is most segmented in the analysis corresponds to those that we have defined as belonging to the digestive-endocrine systems; finally (6) the relationship between back and neck pain and permanent injuries or defects caused by accidents.

Once this first result was obtained, the estimation algorithm of the MGM was modified by introducing higher-order interactions in the graph. Thus, in a second step, the existence of interactions by diagnostic triads was assumed, and a value of k = 3 was set. As the value of k was increased, it could be observed that the density of the network was considerably reduced. The most clearly maintained relationships were as follows: (1) lumbar pain–cervical pain; (2) anxiety–depression; (3) allergy–asthma–respiratory problems; (4) haemorrhoids–constipation; (5) prostate problems–incontinence; (6) osteoarthritis–osteoporosis; (7) heart attack–coronary heart disease; (8) hypertension–diabetes–obesity; (9) hypertension–diabetes [or other heart problems]–cataracts; (10) hypertension–diabetes–cholesterol; (11) osteoarthritis–osteoporosis–cataracts; (12) allergy–hypertension–cataracts; or (13) cataracts–osteoarthritis–allergies, among other weaker relationships. Thus, although in general terms the previously detected structure of interrelationships was maintained, some less intuitive (and so interesting) associations were lost.

**Figure 3:**
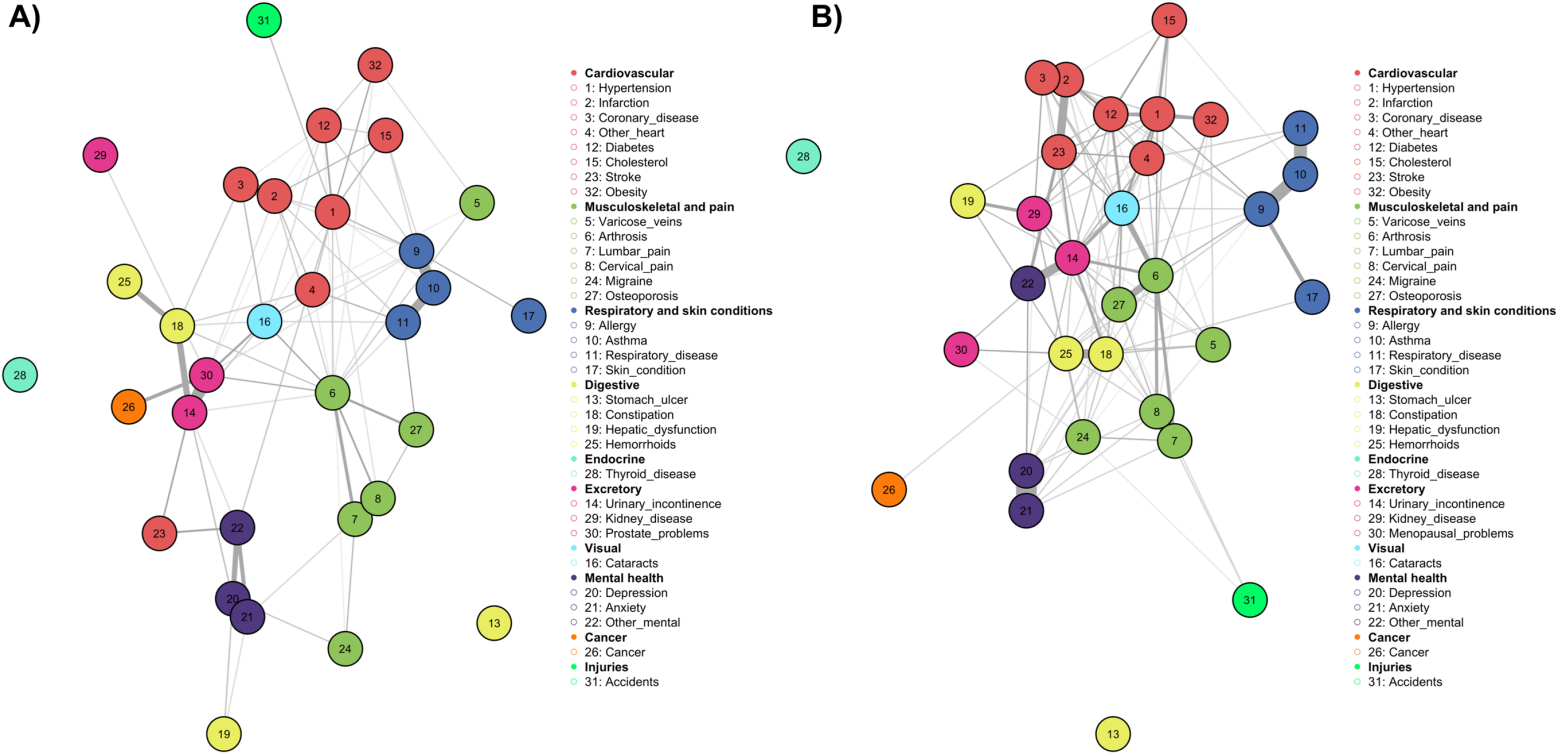
Mixed Graphical Models: (**A**) multimorbidity network using men sample (k = 2, N = 5002); (**B**) multimorbidity network using female sample (k = 2, N = 7281).

Taking into account that certain patterns of multimorbidity might be more prevalent in men or women (e.g. cardiovascular or musculoskeletal conditions, sometimes related to mental health), in a further step the total sample was divided according to the biological sex of the respondent. Figure 3A below shows that for the sample of men (N = 5002), the patterns of multimorbidity were relatively similar to the patterns detected for the full sample. However, we could also identify some relationships between indicators that were men specific. In particular, problems related to the excretory system (prostate problems, incontinence problems, haemorrhoids and constipation) had a clearly defined character in this group. Similarly, the presence of malignant tumours (the variable 'cancer') was significantly related to prostate problems and, for example, stroke appeared as a separate indicator from cardiovascular conditions and, in turn, linked to other mental health problems. In this case, focusing on the male group, we could see that thyroid-related problems and stomach ulcers appeared separately from the other diseases (i.e., were not correlated with other conditions).

Again, with the intention of reducing the density of the resulting graph to explore the broader relationships between the different groups of predictors, we assumed the existence of interactions by diagnostic triads with the value of k = 3. In this case the assumption of higher order interrelationships did not produce a result that would allow us a better reading of the relationships, as there would be a drastic decrease in the overall density of the graph. As was the case in the previous model, some variables were found to have a higher centrality in the association structure. Likewise, we could see that prostate problems had a central and therefore articulating position among the relationships of the predictors related to musculoskeletal problems (arthrosis–lumbar pain–cervical pain), the presence of malignant tumours or other problems related to the excretory apparatus. Similarly, hypertension was found in an intermediate position between other cardiovascular problems, allergies and disability due to accidents, while mental health conditions were represented in isolation in the network. In short, although this graph with third-order interactions yielded some information on the intermediary role of some health conditions, the resulting graph was too sparse to be useful in defining complex multimorbidity patterns.

Once the analysis had been carried out for men, the same process was complete for the sample of women (N = 7281). Although to some extent the resulting graph showed a high degree of similarity to the patterns detected in the men's group, some relationships were observed between indicators linked to the women's profile. Again, problems related to menopause and the excretory system (menopausal problems, incontinence problems, haemorrhoids and constipation) were linked to other mental health problems (i.e., not identified with depression or anxiety). Similarly, the presence of malignant tumours appeared to be linked to constipation and haemorrhoids, or mental health problems and their link to migraines and other musculoskeletal pain, and liver and kidney problems together with mental health problems appeared to have a unique character in the women's group. Finally, as was also observed in the men's group, thyroid-related problems and stomach ulcers appeared separate from the other diseases (Fig. 3B).

The implementation of the model of order k = 3 for women would also result in a low-density graph that would not allow us to identify large groups of relationships. In contrast to the results obtained in the men's group, for women we observed the intermediary role of diseases such as cataracts, hypertension or arthrosis, chronic conditions that were in turn linked. Other relationships were also observed, for example between varicose veins, osteoarthritis and constipation, as well as other mental health problems that were related to urinary incontinence. Among the cardiovascular profile, there was a division between the more serious conditions (heart attack–coronary heart disease) and the more prevalent conditions (obesity–cholesterol–hypertension–diabetes, with the latter two conditions linked to cataracts). The group of variables referring to allergies and respiratory problems were strongly associated, and through allergies were closely linked to hypertension and osteoarthritis.

### Assessing the predictability of MGM

The predictability of the MGM models used for the study and understanding of multimorbidity patterns was assessed. Thus, a comparison was made of the classificatory accuracy (Acc.) obtained from the multimorbidity models that have been used to create the classes of co-occurring chronic diseases observed in the analysis. In particular, the prediction of the resulting models produced satisfactory classification results ranging from 0.63 to 0.97 accuracy values in the case of the male sample. The classification accuracy ratio improved slightly within the female group, with accuracy values ranging from 0.65 to 0.98. In short, values were high enough to consider the general goodness of the models and, above all, taking into account the important variability of the predictors that we had introduced within the same analysis (Table 1).

**Table 1:** Comparison of the classification accuracy of the implemented MGMs (Acc: Accuracy; nAcc: Normalised Accuracy).

Men sample				Woman sample			
ID	Variable	Acc	nAcc	ID	Variable	Acc	nAcc
1	Hypertension	0.631	0.188	1	Hypertension	0.677	0.233
2	Infarction	0.934	0.009	2	Infarction	0.977	− 0.006
3	Coronary disease	0.941	0.000	3	Coronary disease	0.969	0.000
4	Other heart	0.887	0.000	4	Other heart	0.899	0.000
5	Varicose veins	0.878	0.000	5	Varicose veins	0.703	0.017
6	Arthrosis	0.766	0.132	6	Arthrosis	0.713	0.378
7	Lumbar pain	0.812	0.158	7	Lumbar pain	0.750	0.343
8	Cervical pain	0.773	0.290	8	Cervical pain	0.751	0.380
9	Allergy	0.828	0.116	9	Allergy	0.784	0.089
10	Asthma	0.907	0.027	10	Asthma	0.902	0.030
11	Respiratory disease	0.904	0.014	11	Respiratory disease	0.926	0.000
12	Diabetes	0.826	0.000	12	Diabetes	0.867	0.006
13	Stomach ulcer	0.907	0.000	13	Stomach ulcer	0.938	0.000
14	Urinary incontinence	0.919	0.029	14	Urinary incontinence	0.895	0.024
15	Cholesterol	0.630	0.047	15	Cholesterol	0.656	0.055
16	Cataracts	0.815	0.002	16	Cataracts	0.781	0.061
17	Skin condition	0.908	0.000	17	Skin condition	0.904	0.000
18	Constipation	0.960	0.000	18	Constipation	0.885	0.014
19	Hepatic dysfunction	0.973	0.000	19	Hepatic dysfunction	0.983	0.000
20	Depression	0.907	0.228	20	Depression	0.833	0.298
21	Anxiety	0.916	0.085	21	Anxiety	0.838	0.176
22	Other mental	0.966	0.000	22	Other mental	0.967	0.000
23	Stroke	0.951	0.000	23	Stroke	0.970	0.000
24	Migraine	0.903	0.000	24	Migraine	0.790	0.008
25	Haemorrhoids	0.885	0.000	25	Haemorrhoids	0.854	0.009
26	Cancer	0.935	0.000	26	Cancer	0.927	0.000
27	Osteoporosis	0.980	0.000	27	Osteoporosis	0.846	− 0.002
28	Thyroid disease	0.966	0.000	28	Thyroid disease	0.836	0.000
29	Kidney disease	0.908	0.000	29	Kidney disease	0.919	− 0.005
30	Prostate problems	0.825	0.098	30	Menopausal problems	0.913	0.000
31	Accidents	0.845	0.000	31	Accidents	0.913	0.000
32	Obesity	0.781	0.000	32	Obesity	0.795	0.000

In the second column of the table, normalised accuracy (nAcc) indicates, in a scale from 0 to 1, how much one chronic disease can be predicted by all other diseases in the network (i.e., a nAcc. = 1 means that all other nodes perfectly predict the disease). Our results show that the most prevalent diseases are those that can be better predicted by other diseases. Negative values in nAcc indicates that the full model performs worse than the intercept model, a possible overfitting problem in certain diseases. Given the possible overfitting in certain diseases, a cross-validation study was carried out; however, the results did not improve with respect to the model obtained using the EBIC regularization method (see Supplementary Table 1). Moreover, as in this exploratory study we were not looking for a predictive model but for the identification of complex relationships that can be relatively interpretable from a clinical point of view, the results were considered acceptable.

Finally, we analysed the Bootstrap sampling distributions for the male and female groups. For each edge weight resulting from the MGM we presented the proportion of non-zero estimates across all models, obtained for the arithmetic mean of the sampling distribution. Additionally, the specified lower and upper quartiles were chosen, respectively using the 5% (0.05) and 95% (0.95) quartiles. As observed, most of the relationships between nodes in the graphs were set to zero, while the strongest relationships were established by a group of principal nodes that articulated the rest of the relationships in the network models obtained (i.e., those that presented a better characterisation of disease associations).

### Disease centrality analysis

**Figure 4:**
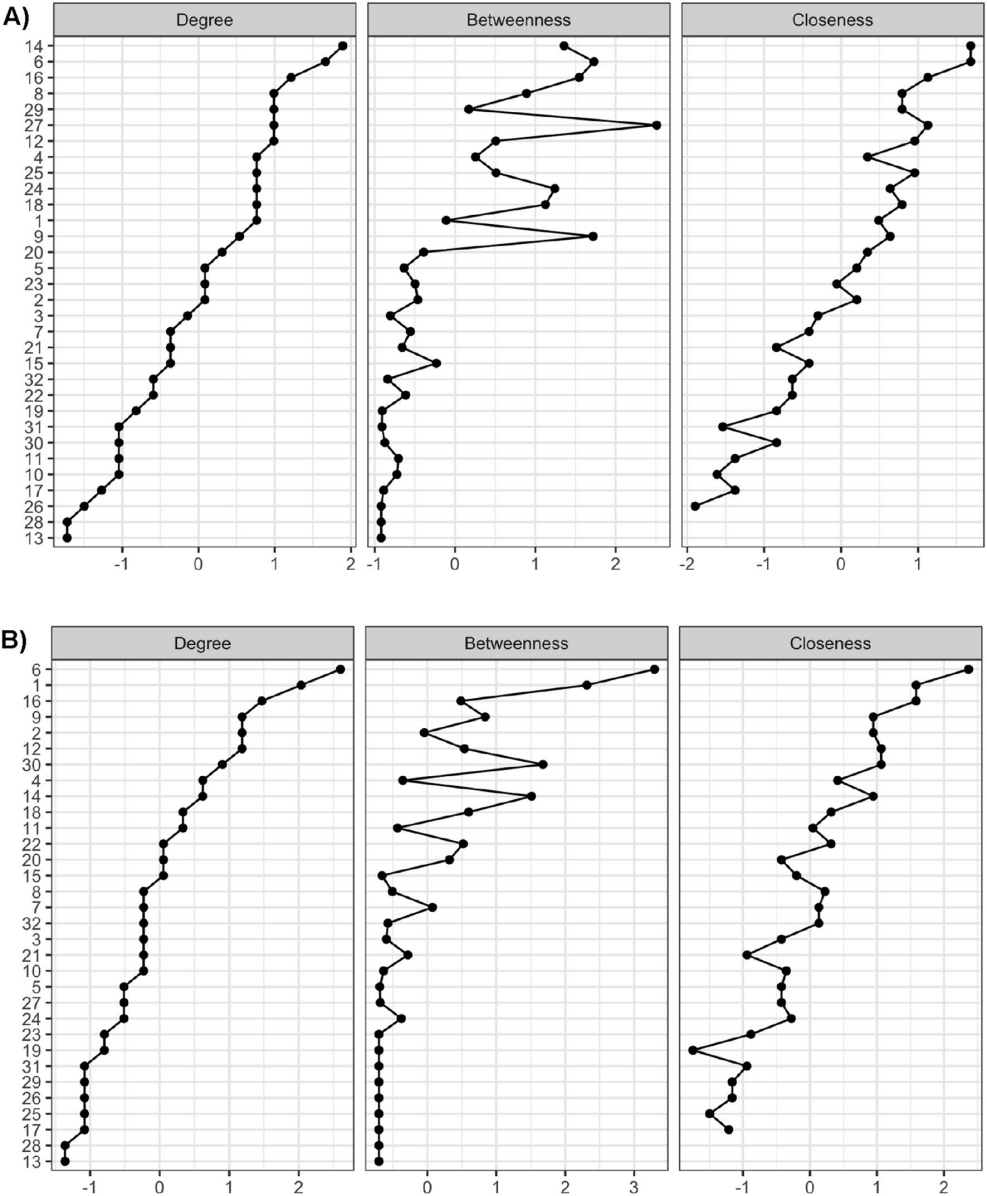
Centrality indexes for males (**A**) and females (**B**) networks (shown as standardized z-scores). Chronic conditions: 1 Hypertension; 2 Infarction; 3 Coronary disease; 4 Other heart; 5 Varicose veins; 6 Arthrosis; 7 Lumbar pain; 8 Cervical pain; 9 Allergy; 10 Asthma; 11 Respiratory disease; 12 Diabetes; 13 Stomach ulcer; 14 Urinary incontinence; 15 Cholesterol; 16 Cataracts; 17 Skin condition; 18 Constipation; 19 Hepatic dysfunction; 20 Depression; 21 Anxiety; 22 Other mental; 23 Stroke; 24 Migraine; 25 Haemorrhoids; 26 Cancer; 27 Osteoporosis; 28 Thyroid disease; 29 Kidney disease; 30 Prostate / Menopausal problems (for males and females, respectively); 31 Accidents; 32 Obesity.

Figure 4 shows three centrality measures—degree, betweenness and closeness—that were used to describe the general relevance of the different chronic diseases in the resulting networks. According the *degree* measure (which describes the number of the connections between variables of interest), we can observe that the variables (i.e., chronic diseases) with higher and lower number of the connections. For men (Fig. 4A), the more relevant diseases were: urinary incontinence (14), arthrosis (6), cataracts (16), cervical pain (8) kidney disease (29), osteoporosis (27), and diabetes (12). In parallel, the closeness centrality index (that quantify indirect connections a node has with other nodes) showed a similar relevance for these variables. However, the betweenness centrality (which measures how important a node is in the average path between two other nodes) described also higher in the z-scores for arthrosis (6), osteoporosis (27) migraine (24), constipation (18), allergy (9), and cholesterol (15) which showed the transversality of these pathologies in this group.

In the case of women (Fig. 4B), the more relevant diseases according the *degree* and *closeness* centrality measures were: arthrosis (6), hypertension (1), cataracts (16), allergy (9), infarction (2), and diabetes (12). However, arthrosis (6), allergy (9), menopausal problems (30), urinary incontinence (14), depression (20), anxiety (21), other mental health problems (22), and lumbar pain (7) presented more transversality in women. These results show that, in principle, less severe conditions such as allergies or arthritis may act as intermediate bonds that make possible the connection between different patterns of multimorbidity that both men and women may suffer.

### Disease community’s identification

Once the best fit of the k = 2 order model was observed, the clustering function of the *igraph* package called *cluster_fast_greedy* was used^49^. Based on the information provided by the resulting networks, this function was oriented towards the identification of dense subgraphs, also called communities in graphs (i.e., networks), by directly optimising a modularity score. In this way, the *cluster_fast_greedy* function calculates the membership vector corresponding to the maximum modularity score, considering all possible community structures along the possible mergers between the different vertices that make up the graph.

**Figure 5:**
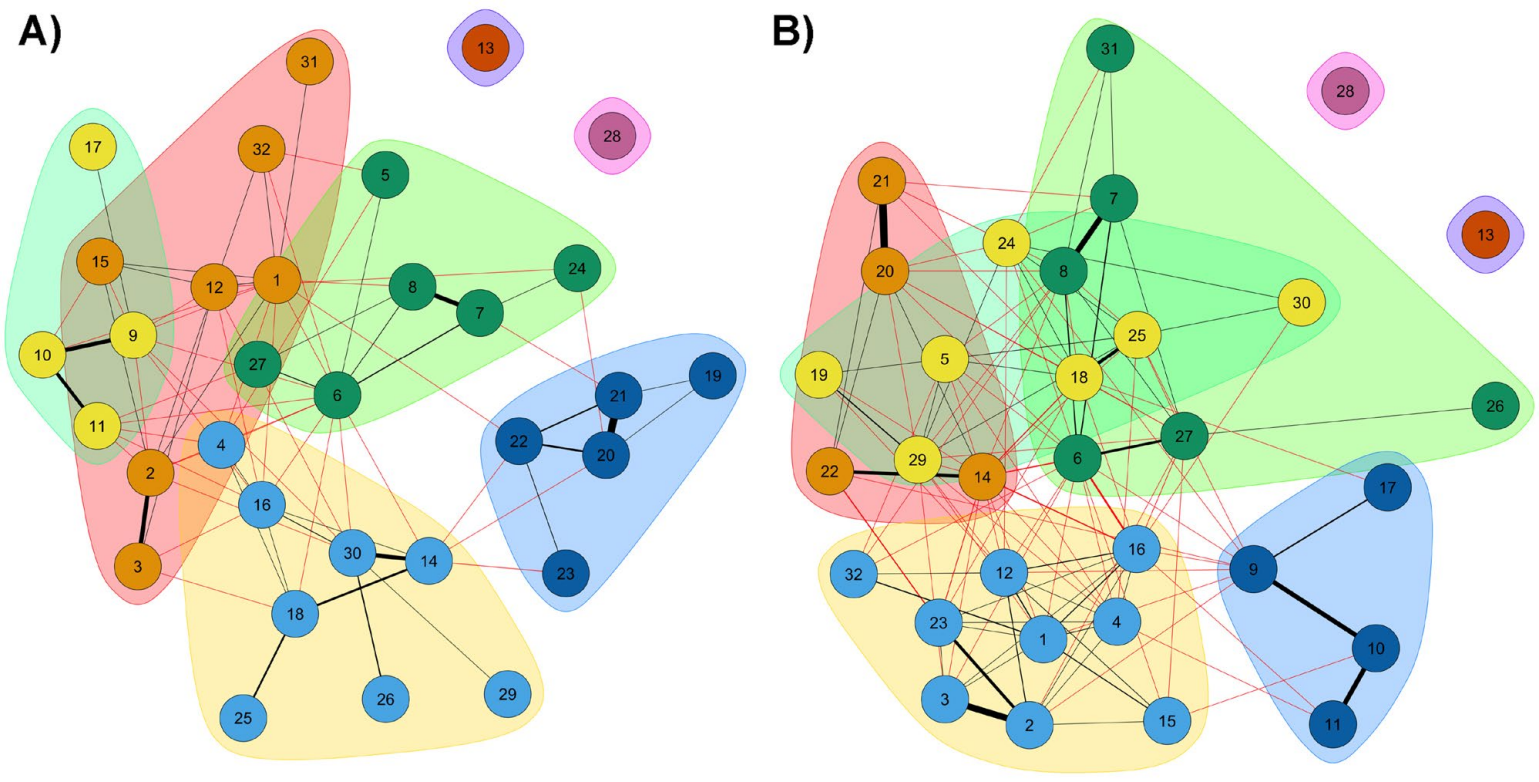
Identification of predictor communities using MGM. (**A**) Men, k = 2; (**B**) Women, k = 2.

As can be seen in Fig. 5A, the community identification analysis allowed to identify a total of seven communities of predictors (or classes) in the men sample, which would be the following: C1 composed of 7 diseases (1, 2, 3, 12, 15, 31, 32: cardiovascular – accidents); C2 composed of 8 diseases (4, 14, 16, 18, 25, 26, 29, 30: combined complex pattern 1); C3 composed of 6 diseases (5, 6, 7, 8, 24, 27: musculoskeletal pattern]; C4 composed of 4 diseases (9, 10, 11, 17: allergies pattern); C5 composed of 5 diseases (19, 20, 21, 22, 23: liver problem – mental health – stroke); C6 composed of 1 disease (13); and C7 composed of 1 disease (28). Figure 5B shows the classification analysis for multimorbidity patterns of women using the community detection analysis. From this analysis we also obtained a set of seven disease clusters, which would be as follows: C1 composed of 4 diseases (20, 21, 22, 14: mental health–incontinence); C2 composed of 9 diseases (1, 2, 3, 4, 12, 15, 16, 23, 32: cardiovascular–cataracts); C3 composed of 6 diseases (6, 7, 8, 26, 27, 31: musculoskeletal–cancer–accidents); C4 made up of 7 diseases (5, 18, 19, 24, 25, 29, 30: digestive system problems–menopausal problems); C5 made up of 4 diseases (9, 10, 11, 17: allergies–respiratory problems–skin problems); C6 made up of 1 disease (13: stomach ulcer); and C7 made up of 1 disease (28: thyroid problems).

In short, for the group of men, seven multimorbidity patterns would be classified as follows: (MP1) characterised by thyroid problems [Thyroid]; (MP2) characterised by stomach ulcer problems [Stomach ulcer]; (MP3) mental health problems [Mental health] (MP4) respiratory problems and allergies [Allergies]; (MP5) cardiovascular and disabling accidents [Cardiovascular]; (MP6) a complex combined character pattern of high prevalence [Pain: musculoskeletal problems associated with pain conditions; (MP7) a complex combined pattern [Complex: other heart problems–urinary incontinence–cataracts–constipation–haemorrhoids–cancer–kidney disease–prostate problems]. On the other hand, for the women's group, seven patterns (but, in this case, better defined) were also identified: (MP1) characterised by thyroid problems [Thyroid]; (MP2) characterised by stomach ulcer problems [Stomach ulcer]; (MP3) mental health problems [Mental health] (MP4) respiratory problems and allergies [Allergies]; (MP5) a pattern of chronic cardiovascular diseases [Cardiovascular], in this case clearly defined; (MP6) a pattern of pain-related musculoskeletal diseases [Pain]; (MP7) a second complex pattern of combined conditions [Complex: varicose veins–liver dysfunction–constipation–haemorrhoids–migraines–kidney disease–menopausal problems].

### Profiling multimorbidity patterns

Ultimately, in an attempt to provide a better characterisation of the social profiles behind the multimorbidity patterns that had been identified in the previous sections, the relationship of these with the social class and age of the individuals was studied. A multinomial regression model was then implemented that would include, in this case, multimorbidity profiles as a categoric dependent variable (Table 2). The social classes (estimated from respondents’ occupation) considered in the study were as follows: (SC1) directors and managers of establishments with 10 or more wage earners and associate professionals; (SC2) directors and managers of establishments with less than 10 wage earners and associate professionals and other technical support professionals; (SC3) intermediate occupations and self-employed; (SC4) supervisors and workers in skilled technical occupations; (SC5) skilled primary sector workers and other semi-skilled workers; and (SC6) unskilled workers.

**Table 2:** Multinomial regression models by gender group.

Pattern	Variables	OR males (CI 95%)	OR females (CI 95%)
MP2	(Intercept)	1.15122 (0.60517–2.1900)	0.21960 (0.14572–0.3309)***
	Age of respondent	1.01213 (1.00055–1.0238)*	1.00826 (1.00090–1.0157)*
	Social class 1	0.89353 (0.50486–1.5814)	0.63857 (0.42241–0.9654)*
	Social class 2	0.78474 (0.43785–1.4065)	0.73356 (0.48311–1.1139)
	Social class 3	1.17768 (0.74577–1.8597)	0.65562 (0.49504–0.8683)**
	Social class 4	1.10867 (0.73395–1.6747)	0.85688 (0.64109–1.1453)
	Social class 5	1.06405 (0.77378–1.4632)	0.88862 (0.70842–1.1147)
	Social class 6	1.18176 (0.71732–1.9469)	0.93904 (0.71173–1.2390)
MP3	(Intercept)	7.16206 (4.01668–12.7705)***	0.99308 (0.77920–1.2657)
	Age of respondent	0.98950 (0.97914–1.0000)*	1.00770 (1.00328–1.0121)***
	Social class 1	1.12687 (0.66794–1.9011)	0.85575 (0.67530–1.0844)
	Social class 2	0.88476 (0.51628–1.5162)	0.97569 (0.76350–1.2468)
	Social class 3	1.98020 (1.30299–3.0094)**	0.78772 (0.66752–0.9296)**
	Social class 4	1.45358 (0.99095–2.1322)*	0.91983 (0.76729–1.1027)
	Social class 5	1.29317 (0.96234–1.7377)*	1.23397 (1.07652–1.4144)**
	Social class 6	1.92992 (1.21950–3.0542)**	1.33029 (1.12366–1.5749)***
MP4	(Intercept)	28.97860 (16.49782–50.9012)***	4.47328 (3.49766–5.7210)***
	Age of respondent	0.96889 (0.95892–0.9790)***	0.97219 (0.96755–0.9769)***
	Social class 1	2.28015 (1.38967–3.7413)**	1.78740 (1.41817–2.2528)***
	Social class 2	1.79097 (1.08130–2.9664)*	1.40056 (1.08647–1.8055)**
	Social class 3	2.08515 (1.38125–3.1478)***	1.20782 (1.01701–1.4344)*
	Social class 4	1.48824 (1.02105–2.1692)*	1.11551 (0.91402–1.3614)
	Social class 5	1.32259 (0.99050–1.7660)*	1.33832 (1.15336–1.5529)***
	Social class 6	1.72898 (1.09669–2.7258)*	0.99098 (0.81158–1.2100)
MP5	(Intercept)	7.05434 (4.03015–12.3479)***	0.41865 (0.32246–0.5435)***
	Age of respondent	1.00256 (0.99245–1.0128)	1.02221 (1.01749–1.0270)***
	Social class 1	1.63255 (1.00031–2.6644)*	0.73503 (0.57563–0.9386)*
	Social class 2	1.01179 (0.61251–1.6713)	1.05211 (0.82510–1.3416)
	Social class 3	1.86874 (1.24704–2.8004)**	0.79944 (0.67724–0.9437)**
	Social class 4	1.36645 (0.94697–1.9717)*	0.83067 (0.69103–0.9985)*
	Social class 5	1.12822 (0.85055–1.4965)	0.96807 (0.84052–1.1150)
	Social class 6	1.48240 (0.94909–2.3154)*	0.84211 (0.70364–1.0078)*
MP6	(Intercept)	0.72536 (0.35036–1.5017)	2.32571 (1.68794–3.2045)***
	Age of respondent	1.01256 (0.99950–1.0258)*	0.96661 (0.96029–0.9730)***
	Social class 1	1.19704 (0.66466–2.1558)	1.63668 (1.21045–2.2130)**
	Social class 2	0.98754 (0.53708–1.8158)	1.29178 (0.92242–1.8090)
	Social class 3	1.12634 (0.68839–1.8429)	1.18312 (0.94035–1.4886)
	Social class 4	0.76290 (0.47754–1.2188)	0.85447 (0.63905–1.1425)
	Social class 5	0.61028 (0.42264–0.8812)**	1.17921 (0.96342–1.4433)
	Social class 6	1.17009 (0.68506–1.9985)	0.92276 (0.69903–1.2181)
MP7	(Intercept)	11.87413 (5.48616–25.7001)***	1.17848 (0.62233–2.2316)
	Age of respondent	0.93544 (0.92025–0.9509)***	0.94540 (0.93130–0.9597)***
	Social class 1	0.98416 (0.39481–2.4533)	1.49017 (0.79077–2.8081)
	Social class 2	1.46415 (0.64033–3.3479)	0.87463 (0.39179–1.9526)
	Social class 3	2.01245 (1.08321–3.7388)*	0.87829 (0.51402–1.5007)
	Social class 4	1.06228 (0.53904–2.0934)	1.08083 (0.60702–1.9245)
	Social class 5	2.06617 (1.32534–3.2211)**	1.02065 (0.65648–1.5868)
	Social class 6	1.86558 (0.93857–3.7082)*	0.93323 (0.51378–1.6951)

Statistically significant coefficients at the level: 0.001***, 0.01**, and 0.05*.

The models yielded some relevant results from the point of view of the individual profiles shaping patterns of multimorbidity. In the case of men, mental health problems seemed to be experienced to a greater extent by young and lower-middle class men. The pattern of allergies was associated with the younger groups, while the cardiovascular pattern was that of upper-middle class elders (i.e. managers and supervisors). The pain pattern was significantly associated with the older population in intermediate occupations and, finally, the complex pattern seemed to be more frequent among the lower classes (i.e. those characterised by manual and unskilled trades). We could also detect some interesting associations in the women group. For example, the pattern of mental health multimorbidity was more frequent among lower-class older women. As in the men's group, allergic profiles were more characteristic of younger, but in this case upper-middle class, groups. Cardiovascular problems were more frequent in older women independently of their social class. The pain pattern was characteristic of young, upper-class women. And finally, the complex pattern did not show statistically significant differences in terms of variations in social class (p > 0.05).

## Discussion

In line with our first objective, it can be said that the use of MGM have enabled a better characterisation and understanding of multimorbidity in Spanish population and, in particular, of the networks of complex interrelationships that can occur within the broad spectrum of chronic pathologies that may coincide in the same person. While machine learning methods are often considered as black-box models through which it is difficult to understand the internal processes of classification^50,51^, this study has shown that the use of graphical mixed models (MGM) can avoid this limitation. Unlike other models in which the potential relationships between the predictors of the models are not clearly visualised, the network models resulting from the MGM analysis made possible a relational reading of the possible latent structures that could be configured from the different chronic diseases that were considered, which was an advantage over the results of other commonly used classificatory models such as those based on principal component analysis, cluster or latent class analysis^52^ which usually overestimate the overall dimensionality and that are hardly interpretable^53^.

According to our second objective, the predictive results of the MGM were satisfactory in terms of goodness of fit. In particular, the prediction of the models produced satisfactory classification results ranging from moderate to high accuracy values (0.63–0.97), and these results were confirmed by bootstrapping methods. Subsequently, the community detection algorithm was used to characterise the different disease communities (or disease neighbourhoods) based on the association discovered from the MGM implementation. From a clinical point of view, compared with previous approaches based on other classification techniques, here we demonstrate how the combined use of community detection algorithms over MGM can provide an interesting alternative for health professionals that need to relationally unravel the complexity of multimorbidity in patients with different social and demographic profiles.

The use of community detection characterised the more relevant multimorbidity patterns for males and females. In the case of men, mental health problems seemed to be experienced to a greater extent by young and lower-middle class men. The pattern of allergies was associated with the younger groups^54–56^, while the cardiovascular pattern was that of upper-middle class elders (i.e., managers and supervisors). The pain pattern was significantly associated with the older population in intermediate occupations and, finally, the complex pattern seemed to be more frequent among the lower classes (i.e., those characterised by manual and unskilled trades). This makes sense considering that the prevalence of multimorbidity tends to be higher in more deprived areas for all age and sex groups^57^. In the case of women, we could also detect some interesting associations. For example, the pattern of mental health multimorbidity was more frequent among lower-class older women. As in the men's group, allergic profiles were more characteristic of younger^54–56^, but in this case upper-middle class groups. Cardiovascular problems were more frequent in older women independently of their social class. The pain pattern was characteristic of young upper-class women. This result that may be linked to the higher prevalence of mental health problems in Spanish women^58–61^. And finally, the complex pattern did not show statistically significant differences in terms of variations in social class (p > 0.05).

In essence, we can summarise the contribution in the following points: (1) some multimorbidity patterns are more prevalent among different socio-economic groups (e.g., allergies are more prevalent among the young and cardiovascular conditions are more prevalent among the older population); (2) the MGM identifies complex multimorbidity profiles that are easily identifiable and interpretable compared to other classical classification techniques, while presenting a more complex and accurate classification of the network of complex interrelationships between different co-occurring diseases; (3) our analysis provides new evidence on the feasibility of MGM models to address the problematic study of the complex combination of co-occurring diseases among large populations characterised by high heterogeneity; (4) it also highlights the need for studies aimed at unravelling complex disease combinations and, in particular, the role of intermediate conditions that may connect different disease patterns which subsequently increase the complexity of medical treatments and the use of health services in specific population groups; (5) evidence that certain chronic diseases tend to occur in combination and interactively, suggesting that a multidimensional and integrative public health strategy is needed to address multimorbidity and its social inequalities^62^.

This research work is not free of limitations. Despite the advantages of the models employed, the high combinatorics generated by the large number of indicators used in this study highlight the need for further work on the progressive refinement of the models and adjustment to specific local context. For example, as the reader will note, some variables could be removed from the analysis in subsequent studies to try to foster a better definition of some of the patterns (for example, by eliminating the distortion of variables that would fall outside the multimorbidity patterns such as stomach ulcers and thyroid disease). Similarly, some of the baseline variables could have been removed from the analysis if we had considered them less relevant. Indeed, having coronary heart disease is not the same as having constipation, or having cancer versus having a stomach ulcer. However, given the exploratory nature of this study, it was considered important not to take any relationship (or lack thereof) for granted. In fact, the models have provided information on many relationships that remain unresolved and will require further analysis. Another limitation of the present work is related to the cross-sectional nature of the study, which prevents us from drawing any conclusions about the patterns of causality that might define the aetiology of the different multi-pathological patterns. For example, for some multimorbidity profiles that have been treated in depth in the literature, such as cardiovascular disease, we can intuit the line of causality (e.g., from obesity to myocardial infarction, passing through other diagnoses such as hypertension, diabetes, etc.), but other patterns are not so clear. In fact, thinking about the link between depression and pain, we might ask what starts first, and we probably could not offer a single answer.

Finally, it should be noted that studies with other definitions of multimorbidity (for example, considering it as 3 or more chronic conditions) could give rise to other patterns of a more serious nature and perhaps better defined from a clinical point of view. In any case, in our case we preferred to adopt a more open definition that, for preventive purposes, would accommodate profiles of lesser severity that would also include multimorbidity in the young population.

Despite these limitations, the results obtained demonstrate the usefulness and feasibility of this approach to the study of multimorbidity patterns based on the use of disease networks, which can offer both researchers and health professionals a holistic, organic and accurate view of the relational structure of large sets of co-occurring chronic diseases (or disease neighbours). Therefore, in terms of the use of methods, the benefits of using a chronic disease network approach for the identification and interpretation of complex multimorbidity profiles are evident, which, with high dimensional and even time series data, could even be used to study the aetiological processes that might determine the emergence and evolution of chronicity in various population groups. However, in addition to the study of the development and co-evolution of chronic disease patterns over time, the existence of differences in multi-pathological profiles associated with socio-economic factors (such as gender, age or social class) opens up a new line of research on social inequalities in multimorbidity that need to be confronted in order to be able to offer an adequate response to the different groups that form part of a complex phenomenon.

Finally, our findings suggest that tailored public health strategies are needed to address social inequalities in multimorbidity in different population strata with different health and social needs, as it is the latter that often determine health outcomes.

## Methods

### Study design

The present study is characterised by a descriptive cross-sectional design, through which is aimed to identify—by implementing mixed graphical models (MGM)—the latent structure of interrelationships between co-occurring chronic diseases that configure the complex patterns of multimorbidity in the Spanish population.

Given the absence of similar studies applied to the field of multimorbidity, we focus our work on the general Spanish population. Although the present study is based on individual-level data, the prevalence analysis of multimorbidity patterns will also be described at the regional level. In this way, we aim to characterise the problem of multimorbidity and its respective patterns (i.e. the classes of co-occurring chronic diseases) in the different geographical areas of the country. Furthermore, this aggregate analysis at the regional level will allow us to measure and contextualise the problem.

### Data source

The study is based on data from the Spanish European Health Survey (SEHS), conducted by the Spanish National Statistics Institute (INE). The SEHS is the Spanish section of the European Health Interview Survey (EHIS), coordinated by Eurostat, the statistical office of the European Commission aimed at harmonising European statistical data and methods, and regulated by Regulation (EC) 1338/2008 and Commission Regulation 141/2013.

The SEHS questionnaire was jointly adapted by the INE and the Ministry of Health with the intention of enabling comparison with the main indicators of the National Health Survey (NHS) by including additional indicators of interest. In this way, the methodology used allows comparison with the data series of the main national indicators, as well as the planning and evaluation of health-related actions from the national to the European level. The SEHS has a five-yearly periodicity aimed at households with stratified tri-stage sampling, where health information is collected regarding the population resident in Spain aged 15 years old and over by means of the implementation of a common European questionnaire consisting of 4 modules: (1) socio-demographic module (household and individual); (2) health status module; (3) health care module; and (4) health determinants module.

Specifically, for this exploratory study oriented towards the application of machine learning techniques and, in particular, of mixed graphical models for the discovery and classification of complex multimorbidity patterns, we will use data from the SEHS 2014, whose fieldwork would be carried out between January 2014 and February 2015, and whose sample would be composed of a total of 22,842 personal interviews. For the present study, taking into account that we were going to work with people with multimorbidity, only those individuals with two or more concurrent chronic diseases were included. In this way, our object of study was adjusted to the theoretical definition of the phenomenon to be studied, while at the same time eliminating possible sources of distortion in the classification that could arise from the inclusion of people with no chronic diseases or with only one diagnosed chronic disease. In short, the final sample would be reduced to a total of 12,283 individuals, which would still provide us with a relevant sample base to answer our study questions.

### Variables of interest

For the study of classes (or patterns) of multimorbidity, indicators referring to diagnosed chronic diseases (objective health condition variables) were selected, including the following: (1) high blood pressure; (2) myocardial infarction; (3) angina pectoris, coronary heart disease; (4) other heart diseases; (5) varicose veins in legs; (6) osteoarthritis (excluding arthritis); (7) chronic back pain (cervical); (8) chronic back pain (lumbar); (9) chronic allergy, rhinitis, conjunctivitis or allergic dermatitis, food allergy or other; (10) asthma (including allergic asthma); (11) chronic bronchitis, emphysema, chronic obstructive pulmonary disease (COPD); (12) diabetes; (13) stomach or duodenal ulcer; (14) urinary incontinence or urine control problems; (15) high cholesterol; (16) high cholesterol; (17) high cholesterol; (18) high blood pressure; (15) high cholesterol; (16) cataracts; (17) chronic skin problems; (18) chronic constipation; (19) cirrhosis, liver dysfunction; (20) depression; (21) chronic anxiety; (22) other mental problems; (23) stroke (embolism, cerebral infarction, cerebral haemorrhage); (24) migraine or frequent headache; (25) haemorrhoids; (26) malignant tumours; (27) osteoporosis; (28) thyroid problems; (29) kidney problems; (30) prostate problems (men only); (31) menopausal problems (women only); (32) permanent injuries or defects caused by an accident. In addition, (33) obesity would be included and estimated from the body mass index. All these variables were binary and were therefore measured in terms of 0 'absence' or 1 'presence' of the chronic, disabling or long-term illness. We also included the sex of the respondent to study differences in multimorbidity patterns.

### Statistical analysis: Mixed Graphical Models

First, the *mgm* package^41^ was used to estimate a *k-order* Mixed Graphical Model (MGM) of multimorbidity patterns based on pairwise associations. This package estimates MGMs using a nodewise estimation method with a penalty based on least absolute shrinkage and selection operator regularization (LASSO)^63^. The visual representation of relationships tends to make it easier to obtain a first impression of the data, however, the interpretation of these graphical models can still be complex when the estimated graphs have a high density of vertices and edges^41^. In order to facilitate the interpretation of the results by reducing spurious correlations, in these models the *glasso* algorithm (i.e., graphical lasso) is a commonly used method to obtain networks with a lower density and ultimately more sparse^64^. The *glasso* algorithm forces low partial correlation coefficients to tend to zero, which favours the sparsity of the networks. Thus, the density of the graph can be controlled by various fitting parameters. The estimation algorithm requires us to make an assumption about the highest order interaction in the graph. The algorithm includes an L1 penalty to obtain a sparse estimate that is controlled by the regularisation parameter lambda (λ), using cross-validation and the extended Bayesian information criterion (EBIC)^41^. EBIC, being more conservative than cross-validation, was used to select an optimal fit of the fitting parameter so that the strongest relationships are retained in the graph (i.e., maximising true positives)^41^. The EBIC itself has a setting parameter gamma (γ), which controls the trade-off between sensitivity and precision, based in a default value of 0.25 (the lower the γ hyperparameter the higher the density of the graph)^65^. The LASSO method generates parsimonious network in which small edges are shrunk to zero, hence only the most robust associations are presented (i.e., using edges or links between nodes), while spurious relationships are excluded and so the likelihood of false positives is reduced^66^.

Given the diversity of chronic conditions analysed in this work and the subsequent complex structure of multimorbidity patterns identified in literature, some relations were expected to be spurious by nature and therefore more likely to be shrunk to zero through this method. To avoid the loss of these relationships the γ value would also be set to 0 (which turns EBIC into BIC), as well as to other higher values that would allow us to identify multimorbidity patterns while losing little information. Between-node predictability was finally calculated for all the binary variables (i.e., chronic diseases) included in the network. The proportion of correct classification was assessed by measures of accuracy (Acc) and normalised accuracy (nAcc).

In a second step, the resulting multimorbidity network were represented using the *qgraph* package^67^. This resulted in an undirected weighted network showing the pairwise associations between the different chronic conditions. An undirected graph *G* = *(V,E)* consists of a collection of nodes or vertices *V* = *{1,2,3,…,p}* and a collection of edges *E ⊂ V* × *V.* In MGM the graphical model *G* is then obtained from the parameter estimates. Therefore, the resulting MGM consists of a set of elements or variables (i.e., chronic conditions), represented by circles, and a set of lines visualising the relationships between the elements or variables^40,45^. The thickness of these lines represents the strength of the relationships between the items or variables; conversely, the absence of a line implies that there are no relationships or that they are too weak to be relevant. In particular, in the MGM, these lines capture partial correlations, i.e. the correlation between pairs of variables when controlling for all other associations in the dataset, which has the advantage of avoiding spurious correlations.

After this step, the resulting network structure was described through three main centrality measures using the *centralityPlot* function^67^: degree (measures the number of the connections between variables of interest), closeness (quantify indirect connections a node has with other nodes), and betweenness centrality (describes how important a node is in the average path between two other nodes)^68^. Once the best fit of the k = 2 order model was observed and we had a deep description of the different possible multimorbidity patterns and their central chronic conditions, the clustering function of the *igraph* package called *cluster_fast_greedy* was used^46^. Based on the information provided by the networks we have, this function was oriented towards the identification of dense subgraphs, also called communities in graphs (i.e., networks), by directly optimising a modularity score. In this way, the *cluster_fast_greedy* function calculates the membership vector corresponding to the maximum modularity score, considering all possible community structures along the possible mergers between the different vertices that make up the graph. This function was be used to identify and extract multimorbidity patterns (or clusters) that would be subsequently characterised by a multinomial regression analysis which included age of respondent and (occupation-based) social class as predictors.

Finally, bootstrapping methods were used to assess the accuracy of the estimated network. The resample function implemented in the R-package mgm was used and to analyse the robustness of our network obtained using γ = 0, the same estimation procedure was repeated with the default value of γ = 0.25 and γ = 0.50, and estimations of the three networks were compared^41^. For each edge weight resulting from the MGM we show the proportion of non-zero estimates across all models, obtained for the arithmetic mean of the sampling distribution. Additionally, the specified lower and upper quartiles are shown. In our case, we chose the 5% (0.05) and 95% (0.95) quartiles. Then, we calculated 100 bootstrapped samples and plotted the resulting sampling distributions of all possible associations for the male and female groups, representing the 5% (0.05) and 95% (0.95) quantiles of the sampling distribution and the proportion of non-zero estimates across all models.

## Supplementary Information

Supplementary Table 1.

## Data Availability

The dataset used and analysed during the current study is publicly available and can be freely downloaded from the Spanish National Statistics Institute.
