# Supplementary material for: Discovery and classification of complex multimorbidity patterns: unravelling chronicity networks and their social profiles
Source: Sci Rep. 2022 Nov 21;12:20004. doi: 10.1038/s41598-022-23617-8 (PMC9678882; doi:10.1038/s41598-022-23617-8)
Supplement: Supplementary file 1 — Supplementary Table 1. [file 41598_2022_23617_MOESM1_ESM.docx]

**Supplementary table 1. Cross-validation (lambda=10)**

| **Men sample** | **Women sample** |
| --- | --- |
| **Variable CC nCC** | **Variable CC nCC** |
| 1 Hypertension 0.628 0.181 | 1 Hypertension 0.681 0.243 |
| 2 Infarction 0.935 0.021 | 2 Infarction 0.978 0.000 |
| 3 Coronary disease 0.942 0.003 | 3 Coronary disease 0.968 -0.022 |
| 4 Other heart 0.887 0.002 | 4 Other heart 0.900 0.011 |
| 5 Varicose veins 0.878 0.000 | 5 Varicose veins 0.711 0.042 |
| 6 Arthrosis 0.771 0.150 | 6 Arthrosis 0.713 0.380 |
| 7 Lumbar pain 0.811 0.152 | 7 Lumbar pain 0.752 0.348 |
| 8 Cervical pain 0.772 0.287 | 8 Cervical pain 0.748 0.372 |
| 9 Allergy 0.828 0.115 | 9 Allergy 0.784 0.089 |
| 10 Asthma 0.907 0.025 | 10 Asthma 0.901 0.028 |
| 11 Respiratory disease 0.907 0.049 | 11 Respiratory disease 0.927 0.020 |
| 12 Diabetes 0.826 0.001 | 12 Diabetes 0.869 0.019 |
| 13 Stomach ulcer 0.907 0.000 | 13 Stomach ulcer 0.938 0.000 |
| 14 Urinary incontinence 0.921 0.050 | 14 Urinary incontinence 0.896 0.031 |
| 15 Cholesterol 0.632 0.053 | 15 Cholesterol 0.665 0.079 |
| 16 Cataracts 0.815 0.004 | 16 Cataracts 0.790 0.100 |
| 17 Skin condition 0.908 0.000 | 17 Skin condition 0.904 0.000 |
| 18 Constipation 0.959 -0.015 | 18 Constipation 0.885 0.020 |
| 19 Hepatic dysfunction 0.973 0.000 | 19 Hepatic dysfunction 0.983 0.000 |
| 20 Depression 0.907 0.228 | 20 Depression 0.828 0.278 |
| 21 Anxiety 0.921 0.137 | 21 Anxiety 0.841 0.193 |
| 22 Other mental 0.966 0.000 | 22 Other mental 0.968 0.004 |
| 23 Stroke 0.952 0.016 | 23 Stroke 0.970 -0.018 |
| 24 Migraine 0.903 0.000 | 24 Migraine 0.791 0.014 |
| 25 Haemorrhoids 0.886 0.014 | 25 Haemorrhoids 0.855 0.011 |
| 26 Cancer 0.935 -0.003 | 26 Cancer 0.927 0.000 |
| 27 Osteoporosis 0.980 0.000 | 27 Osteoporosis 0.846 -0.002 |
| 28 Thyroid disease 0.966 0.000 | 28 Thyroid disease 0.836 0.000 |
| 29 Kidney disease 0.909 0.002 | 29 Kidney disease 0.919 -0.005 |
| 30 Prostate problems 0.826 0.103 | 30 Menopausal problems 0.913 0.000 |
| 31 Accidents 0.845 0.000 | 31 Accidents 0.913 0.000 |
| 32 Obesity 0.781 -0.001 | 32 Obesity 0.797 0.008 |
